# Platelets as Key Factors in Inflammation: Focus on CD40L/CD40

**DOI:** 10.3389/fimmu.2022.825892

**Published:** 2022-02-03

**Authors:** Fabrice Cognasse, Anne Claire Duchez, Estelle Audoux, Theo Ebermeyer, Charles Antoine Arthaud, Amelie Prier, Marie Ange Eyraud, Patrick Mismetti, Olivier Garraud, Laurent Bertoletti, Hind Hamzeh-Cognasse

**Affiliations:** ^1^ Etablissement Français du Sang Auvergne-Rhône-Alpes, Saint-Etienne, France; ^2^ SAINBIOSE, INSERM, U1059, University of Lyon, Saint-Etienne, France; ^3^ Vascular and Therapeutic Medicine Department, Saint-Etienne University Hospital Center, Saint-Etienne, France

**Keywords:** platelets, innate immunity, transfusion, cytokine/chemokine, inflammation, CD40L/CD40 pathway

## Abstract

Platelets are anucleate cytoplasmic fragments derived from the fragmentation of medullary megakaryocytes. Activated platelets adhere to the damaged endothelium by means of glycoproteins on their surface, forming the platelet plug. Activated platelets can also secrete the contents of their granules, notably the growth factors contained in the α-granules, which are involved in platelet aggregation and maintain endothelial activation, but also contribute to vascular repair and angiogenesis. Platelets also have a major inflammatory and immune function in antibacterial defence, essentially through their Toll-like Receptors (TLRs) and Sialic acid-binding immunoglobulin-type lectin (SIGLEC). Platelet activation also contributes to the extensive release of anti- or pro-inflammatory mediators such as IL-1β, RANTES (Regulated on Activation, Normal T Expressed and Secreted) or CD154, also known as the CD40-ligand. Platelets are involved in the direct activation of immune cells, polynuclear neutrophils (PNNs) and dendritic cells *via* the CD40L/CD40 complex. As a general rule, all of the studies presented in this review show that platelets are capable of covering most of the stages of inflammation, primarily through the CD40L/CD40 interaction, thus confirming their own role in this pathophysiological condition.

## Introduction

Blood platelets are anucleate cells produced by the fragmentation of megakaryocytes (MK). These small, disc-shaped cells circulate at a rate of 150,000 to 350,000 platelets per μl of blood. They have a life span of 7-10 days. Following injury to the vascular wall, they interact very quickly with the subendothelium and are activated to prevent bleeding. They lose their discoid shape and become spherical, emitting long filopodia to facilitate their adhesion. They then spread and aggregate, forming a platelet plug to stop bleeding from capillaries and small vessels (1). The study of platelet morphology by electron microscopy has highlighted three major components: i) the plasma membrane with an open canalicular system (OCS), ii) the cytoskeleton and iii) the various intracellular organelles (1). The OCS corresponds to deep invaginations of the plasma membrane and runs continuously along the cell surface. It contributes to the uptake of external elements into the platelets and to the release of granule contents upon activation. This system serves mainly as a membrane reservoir as the platelets change shape (1, 2).

The plasma membrane is supported by a highly developed cytoskeleton, consisting of microtubules and actin microfilaments (1). It plays an important role in platelet biology and, more specifically, in maintaining and changing the shape of platelets during activation and aggregation. It also helps to release the contents of the granules. Platelet cytoplasm contains numerous organelles, in particular some mitochondria, glycogen grains, alpha and dense granules and lysosomes. During platelet activation, the contents of the granules are secreted and a number of granule-specific proteins are detected on the platelet surface. They are used as secretion markers in flow cytometry (3–5).

## Platelet Functions

Haemostasis is a physiological process that maintains the fluidity of the blood and prevents bleeding during vascular injury. The key role of platelets in haemostasis and thrombosis has been documented for many years (6, 7). Multiple therapeutic targets have been identified and used in the development of anti-thrombotic drugs. However, work published in recent years shows that the role of platelets is not confined to maintaining vascular integrity and thrombosis as they play an important role in inflammation, cancer dissemination, wound healing and the separation of blood and lymphatic vessels during development. Platelets impact inflammation and the innate immune response on several levels (8–14). They express toll-like receptors (TLRs) involved in the innate immune response and may thereby contribute to the response to infections by secreting a number of inflammatory mediators (8, 13, 15–17). Furthermore, several receptors on the platelet surface recognise ligands present on monocytes and neutrophils, resulting in the formation of circulating leuko-platelet aggregates (14). Activated platelets can secrete chemokines that contribute to monocyte recruitment (18–20) or macrophage differentiation (21). The interaction of platelets with neutrophils *via* PSGL-1, a P-selectin ligand, is key in initiating the innate inflammatory response and neutrophil extravasation (22–24). Platelet interactions with endothelial cells during infection also condition monocyte migration to the site of inflammation (25).

The CD40 ligand (CD40L) produced by platelets induces an inflammatory response in the endothelium (26–29). Indeed, CD40L can cause endothelial cells to produce reactive oxygen species and express adhesion molecules, chemokines and tissue factor (26, 29). Unlike CD40L, which is stored in platelets, IL-1β is synthesised during platelet activation (30, 31). IL-1β production is sufficient to induce endothelial cells to express genes involved in leukocyte adhesion (32). IL-1β activates endothelial cells causing increases in chemokine secretion and in the expression of molecules that trigger neutrophil and monocyte adhesion to the endothelium (25).

Thus, the involvement of platelets in inflammation is a dynamic process involving various mechanisms. It is important to note that this phenomenon is reciprocal since reference has been made to the pleiotropic role of leukocytes in haemostasis and thrombosis known as “immunothrombosis” (33). The importance of the intimate relationship between platelets and inflammatory cells is found in several diseases, including neurodegenerative diseases, atherosclerosis, acute coronary syndrome, rheumatoid arthritis and lupus (13, 34–37).

## Platelet Granules

Correct platelet function is largely determined by soluble factors that are secreted by alpha and dense granules as well as by lysosomes (38–42). Indeed, platelets store small molecules in their dense granules and several hundred different proteins in their alpha granules. The granules are generated in the megakaryocytes and/or collected in the circulating plasma (like a sponge) by mechanisms that are still not fully understood.

### Alpha Granules

In platelets, alpha granules account for approximately 10% of the platelet volume and are present at a rate of 50 to 80 alpha granules per platelet. They are spherical or oval in shape, and 200-500 nm in diameter (43). These granules contain a wide variety of adhesive proteins that are important for primary haemostasis, such as von Willebrand factor (vWF), fibrinogen, fibronectin, vitronectin and thrombospondin (41). These proteins are important to the adhesive properties of platelets and the formation of a stable thrombus. Alpha granules also contain molecules that play a role in coagulation (factors V, XI, XIII) as well as in wound healing, inflammation and angiogenesis (41). These molecules include PF4 (platelet factor 4), IL-8 (interleukin 8), PDGF (platelet derived growth factor), TGF-β (transforming growth factor-β) and VEGF (vascular endothelial growth factor) (41). PF4, β-thromboglobulin (or CXCL7) and Rantes (or CCL5) are the most abundant chemokines in the alpha granules. Some platelet alpha granule components, such as fibrinogen or albumin, are internalised while others are synthesised in the megakaryocyte and then loaded into the granules (2). The theory that alpha granules represent a homogeneous population of organelles has been challenged by several groups. Italiano’s team in particular showed that pro- and anti-angiogenic proteins are distributed to varying degrees in the alpha granules and are secreted differently and independently (44–46).

### Dense Granules

Dense granules (or δ granules) are found solely in platelets (47–49). These granules are fewer in number (3-8 per platelet) and smaller (100-300 nm in diameter) than the alpha granules (50). They contain high concentrations of cations, polyphosphates, ADP, ATP, serotonin, calcium and polyphosphates, giving them an opaque appearance on examination under an electron microscope (47). Their membranes contain the lysosome markers LAMP2 and CD63 but not LAMP1 (47–53).

Despite their relevance for haemostasis, inflammation and thrombosis, the biogenesis of alpha and dense granules has not been entirely elucidated.

### Granule Release Mechanisms

Exocytosis of platelet granule contents occurs by mechanisms similar to those used by other secretory cells such as neurons. Exocytosis involves reorganisation of the cytoskeleton, movement of the granules to the plasma membrane, fusion between the plasma membrane and the granule membrane and the release of the intracellular contents (3, 40, 54, 55).

Phospholipase C and G-protein-coupled receptor signalling pathways activate different isoforms of Protein kinase C which, in turn, phosphorylate several molecules involved in exocytosis. Although the mechanism of platelet secretion has not been explained in full, it is thought to be similar to that of neurons. Indeed, the active secretory machinery comprises the soluble N-ethylmaleimide sensitive factor(NSF) in the nucleus and soluble NSF-associated protein receptor complexes (SNAREs) located on the surface of vesicles (vSNAREs, vesicular SNAREs) and target membranes (tSNARES, target membrane SNAREs) (3, 5, 40, 55, 56). The interaction between vSNAREs and tSNAREs forms a stable complex that brings the two membranes into close proximity and prompts their fusion. Platelets express SNAREs proteins, including (i) vSNAREs which correspond to vesicle-associated membrane proteins (VAMPs) -2, -3 and -8 (ii) and tSNAREs which include soluble NSF-associated proteins (SNAPs) -23, -25 and -29 and syntaxins 2, 4, 7 and 11 (3, 57, 58).

Similarly, platelets express SNARE regulatory proteins such as Sec1 and Rab. Under basal conditions, the platelet Sec1 protein (PSP) prevents SNARE complex formation by binding to tSNAREs, thereby inhibiting membrane-vesicle fusion. Following platelet activation and PKC activation, the platelet Sec1 protein is phosphorylated and NSF is activated.At the same time, Rab GTPase (Rab27a) links with Munc13-4 and SLP4 (Synaptotagminlike protein 4), attaching the granules to the OCS or plasma membrane. This interaction and assembly process triggers vSNARE-tSNARE association and facilitates vesicular attachment to the OCS, forming a SNARE complex that precedes membrane fusion. Finally, NSF modulates SNARE conformation in a zipper-like manner, causing granule fusion with the OCS and releasing the contents of the granules (5, 57, 59–64).

## CD40L

CD40L, also known as CD154, is a type II transmembrane protein belonging to the TNF (Tumour Necrosis Factor) superfamily. This protein was originally identified as a ligand of the CD40 receptor with a molecular weight ranging from 32 to 39 kDa due to post-translational changes. Since the CD40L-CD40 axis was first discovered in immune cells, the first signals to be identified were immunological in nature (65–67). Numerous additional studies have revealed the expression of the CD40L-CD40 axis in non-immune cells and the presence of other CD40L receptors such as αIIbβ3, α5β and Mac-1. These studies led to the discovery of the thrombo-inflammatory role of CD40L by ensuring the interaction between platelets and the immune system (9, 28, 66, 68–70).

CD40L is expressed by several haematopoietic and non-haematopoietic cells such as B cells, activated T cells, macrophages, neutrophils, dendritic cells (DCs), activated platelets, endothelial cells and smooth muscle cells. In contrast to neutrophils, cells constitutively expressing CD40L, such as B lymphocytes, macrophages and DCs, increase the expression of this ligand in response to cytokines (71–78). Four CD40L receptors have been identified to date, namely the classical CD40 receptor and the three integrins: αIIbβ3, α5β1 and αMβ2 or Mac-1 (29). However, only CD40, αIIbβ3 and α5β are expressed on platelets.

While CD40L is intracellularly localised in inactivated platelets, activated platelets express CD40L on their surface, allowing them to interact with CD40-expressing cells such as other platelets or endothelial cells, in addition to lymphocytes and DCs. Platelet-derived CD40L has been shown to induce the differentiation of monocytes into DCs and their maturation, in addition to positively regulating the expression of their costimulatory molecules (71–75, 78). This function of platelet-derived CD40L may be highly relevant for systemic lupus erythematosus (SLE), an autoimmune disease in which platelets have been shown to induce DC differentiation and IFN release, thereby promoting antibody secretion by B lymphocytes (79–82). Platelets expressing CD40L have also been identified in different diseases where they directly activate the endothelium (26, 28, 68, 83) or contribute to the recruitment of neutrophils and T lymphocytes to the damaged endothelium, especially in the intima and plaques in atherosclerosis. Thus, in accordance with *in vivo* and *in vitro* investigations that have highlighted the role of platelet CD40L in B lymphocyte isotype switching and increased CD8+ T lymphocyte function during infection, these multiple studies suggest that platelets, *via* CD40L, may impact on lymphocytes and DCs in key stages of adaptive immunity (18, 84–86).

## Involvement of the CD40L/CD40 Axis in Platelet Functions

### In the Context of Thrombo-Inflammation

Henn and colleagues (26, 87) were the first to attribute a physiological modulatory role to the CD40L/CD40 axis showing that it temporally regulates undesirable inflammation during thrombogenesis. In particular, they demonstrated that platelets activated by an agonist express CD40L which triggers an inflammatory response in CD40 expressing endothelial cells. Increased aggregation then allows platelet interactions between CD40L and CD40, which leads within a few minutes to a few hours to the cleavage of CD40L (87), which becomes soluble sCD40L. Langer et al. (88) showed that platelet CD40 has a primary haemostatic role. The work of Danese et al. (27, 89–93) was among the first to demonstrate the direct inflammatory effect of platelets *via* the CD40L/CD40 axis. They showed that lymphocytes expressing CD40L activate platelet CD40, which triggers the release of RANTES (CCL5) that binds to endothelial cells to enhance T lymphocyte recruitment (89, 92, 93). Similarly, Inwald et al. showed that the binding of sCD40L to CD40 induces P-selectin expression and partially activates αIIbβ3 without inducing platelet aggregation (94, 95). Moreover, Gerdes and colleagues (96–99) showed that platelet CD40 is involved in atherosclerosis by triggering platelet-leukocyte aggregate formation and endothelial cell activation.

There is considerable evidence to suggest that platelet transcription factors play a key role in regulating inflammatory and haemostatic functions (100). Yacoub et al. (101, 102) demonstrated that platelets express TRAF-1, -2 and -6 and that binding of sCD40L induces recruitment of TRAF-2 to CD40. The sCD40L/CD40/TRAF2 axis was also shown to induce activation of Rac1 and p38 MAPK, leading to P-selectin expression and a change in platelet shape (103). In addition, this same axis allowed for increased platelet aggregation *ex vivo* and thrombus formation *in vivo* in the presence of suboptimal doses of platelet agonists. Hachem et al. (103) showed that sCD40L induces phosphorylation of IkBα, the inhibitory subunit of NF-κB which, unlike nucleated cells, is involved in elusive functions not affecting the genome. The activation of NF-κB induced by sCD40L was also found to be independent of the sCD40L/CD40/TRAF2/Rac1/p38 MAPK axis and essential for the expression of P-selectin as well as for the potentiation of platelet aggregation in response to low doses of platelet agonists (103). The study conducted by Kojok and colleagues (104) then demonstrated that both the activation of NF-κB induced by sCD40L and the potentiation of aggregation occur exclusively downstream from the activation of the CD40 receptor. A second study by the same research group (105) demonstrated that acetylsalicylic acid (ASA) reduces sCD40L-enhanced platelet TxA2 secretion and sCD40L-potentiated platelet aggregation by inhibiting platelet agonist-induced phosphorylation of the Myosin Light Chain. It should be noted that the two platelet priming axes activated by the sCD40L/CD40 axis (p38 MAPK and NF-κB) were not affected by ASA (105). This indicates that sCD40L may be responsible for the reduced efficacy of regular antiplatelet therapy in some patients with coronary artery disease (105). These studies conducted by Merhi and colleagues (102, 104) contradict an earlier study by André et al. (106–108) which showed that the sCD40L/CD40 axis is not thrombogenic since thrombus formation in CD40-/- mice is intact, in contrast to CD40L-/- mice which develop delayed thrombogenesis. In parallel, they also showed that sCD40L binds to αIIbβ3 as strongly as fibrinogen, and that this binding is more likely to stimulate platelet activation. However, the mechanisms involved in platelet activation by the sCD40L/αIIbβ3 axis have not been sufficiently explained. These contradictions may suggest that CD40 and αIIbβ3 are, respectively, the high and low affinity receptors for sCD40L.

Data have implicated sCD40L in endothelial dysfunction and angiogenesis. Napoleao et al. showed that the concentration of sCD40L increased over time after the onset of myocardial infarction, associated with endothelial NOS (eNOS) polymorphism and Vascular Endothelial Growth Factor (VEGF) concentration (109). These results indicate that sCD40L should not only be considered involved in haemostasis and inflammation, but also plays an important role in vascular and endothelial dysfunction (109–113). Although most studies confirm the important role of the various platelet sCD40L/CD40 axes in thrombo-inflammation, further studies are needed to describe their complete signalling pathways and the precise molecular effects they have on platelet functions.

### In an Infectious Context

The role of platelets is not limited to the haemostatic response. In an infectious context, platelets express many members of the TLR family. TLRs 1-10 and their signalling pathways, adaptor molecules and transcription factors have been identified in humans and are all expressed by platelets (8, 15–17, 114–119). These TLRs allow the platelet to play a critical role in innate immunity through the recognition of PAMPs and DAMPs. TLR 4 recognises LPS, a major component of the wall of Gram-negative bacteria (17, 117, 118, 120). The binding of LPS by TLR 4 is not direct, but involves two accessory plasma proteins, namely LBP-LPS binding protein and CD14. CD14 is present on the surface of cells, but not on the surface of platelets - hence its soluble form (sCD14) present in blood allows interaction with two TLR 4-MD-2 (myeloid differentiation factor 2) complexes and leads to their dimerization (121–124). Human platelets can discern various isoforms of bacterial LPS *via* TLR 4, resulting in distinct cytokine secretion profiles and a modulated response to different pathogen species (125, 126). Platelets in contact with LPS are thought to play a significant role in the production of TNF-α (116), TF (127) and sCD40L (128) in addition to increasing the phagocytosis of platelets linked to auto-antibodies (129). Furthermore, it was observed that septic patients with thrombocytopenia had higher levels of TLR4 expressed on the surface of platelets and more sCD40L in the bloodstream, indicating high levels of blood platelet activation (130).

Stimulation of human platelet-rich plasma (PRP) by *R. africae* (and also stimulation of TLR2 in PRP by its synthetic ligand, Pam3Cys) has been shown to increase sCD40L levels, whereas the use of TLR2-blocking antibodies could block this effect (131). As activated platelets are considered the principal source of sCD40L (93, 106), these effects result from TLR2-mediated platelet activation. Several studies characterise the role of platelet TLR2 in infections due to periodontal pathogens such as *A. actinomycetemcomitans* and *P. gingivalis*, demonstrating positive, TLR2-dependent regulation of CD40L membrane expression on the platelet surface (122, 132). Our team also demonstrated that the increased release of sCD40L from platelets was mediated by TLR2 and NF-kB signalling (133).

Platelets also act as immune cells when it comes to binding and internalizing viruses (8, 9, 16, 134).

Morris Madzime et al. have published a review (135) and discussed, the role of neutrophils and platelets in HIV transmission and disease. They have also considered the impact of HIV and the most commonly used antiretroviral drugs on the number and function of both cell types, as well as on their interactions with each other. They have the importance of the pro-adhesive interactions between platelets and neutrophils involving CD40 and ICAM-2, on the former, and CD40L and LFA-1 on the latter. Authors suggest that HIV infection induces platelet activation that fuels neutrophil activation, adhesion, endothelial transmigration and NET formation. Moreover, studies have focused on the potential for primary interactions between platelets and HIV env proteins (136) and the secretory response of healthy platelets upon exposure to recombinant HIV-1MN gp120 or gp41 peptides has been investigated. Indeed, in the presence of two out of ten peptides, there was a small but significant decrease in RANTES production, which was restored by using blocking monoclonal antibodies to gp41. It has also been shown that, despite the fact that hyperresponsive, stimulated platelets from HIV-infected patients with HAART release moderate amounts of RANTES and no additional sCD62P, they retain their ability to release additional sCD40L and GRO-alpha, which may aid immune system activation (137). Interestingly, Daniele Pastori et al. have described that increased platelet oxidative stress is associated with HIV-1 infection, related to NOX2 activation. Platelet NOX2 activation plasma and HIV-1 infection were correlated with sCD40L levels *in vivo* suggesting an association between HIV-1 infection, platelet oxidative stress and platelet activation (138).

Recently, several studies uncovered the role of platelet dysfunction in inflammation associated with severe COVID-19 patients and the potential underlying mechanisms leading to this event. Although patients with severe COVID-19 present several clinical manifestations, thromboembolic events constitute a common significant cause of morbidity and mortality in patients infected. Severe COVID-19 is also associated with hyperinflammation, especially in pre-existing cardiovascular disease. Platelet dysfunction is frequently observed in COVID-19 patients, indicating a loss of homeostasis in platelet function, vascular integrity and the induction of coagulation cascade. Annabel Blasi et al. (139) have showed that plasma levels of activated factor VII were lower in COVID patients while levels of the platelet activation marker soluble CD40 ligand were similar in patients and controls. In contrast, Hind Hamzeh-Cognasse, et al. (83) have described that the CD40L plasma level was significantly elevated in the early stages of the disease. Interestingly they observed that the soluble CD40L plasma level decreased overtime while that of sCD62P increased significantly, highlighting the importance of the inflammatory kinetics in COVID-19. Finally, Tianyang Li et al. (140) have defined a mechanism that may be a potential therapeutic target in severe COVID-19. They showed that hypercoagulation and the cytokine storm in severe COVID-19 were linked through the Spike-CD42b interaction that activates platelets, the CD40L-CD40 and the P-selectin-PSGL-1 interactions that bind them to monocytes, and the strong induction of IL-1β in monocytes.

### In a Transfusion Context

sCD40L is mainly produced by platelets after their activation. Its concentration is increased during the storage of platelet concentrates. Studies have reported that sCD40L levels can be linked to genetic markers. Indeed, our team has identified polymorphism in the ITGA2 gene (coding region of the platelet collagen receptor), which is associated with a significant change in sCD40L secretion (141).

Platelet storage lesions include the appearance of platelet activation markers, morphological changes, mitochondrial dysfunction, loss of GPIbα expression and α-granule secretion (142). The storage of platelet concentrates can lead to the secretion of several BRMs (Biological response modifiers), such as sCD40L, PDGFAA, RANTES, IL1β, IL6, IL7, IL8, PF4, IL13, OX40L, IL27 and TGFβ (9, 70, 143). Generally, prolonged storage of platelet concentrates is accompanied by increased BRM production. This may be related to an increase in the percentage of Recipient Adverse Events (RAEs) observed as a function of the platelet concentrate storage period. Platelet concentrates should preferably be transfused as early as possible in order to limit RAEs. However, this conclusion should be considered in line with the constraints of platelet concentrate production and delivery by blood banks, and the demand for products by health care institutions. In particular, from the third day of storage of platelet concentrates, our team highlighted a significant increase in the concentration of these BRMs, particularly sCD40L (144). These observations suggest that storage lesions play a role in the inflammation induced by platelet concentrates. Indeed, sCD40L triggers the production of Reactive Oxygen Species (ROS) during the storage of platelet concentrates, leading to an increase in the production and release of proinflammatory molecules (145).

Storage damage triggered by extrinsic (preparation methods) or intrinsic (plasma and platelet factors, residual leukocytes) factors could be largely responsible for a decrease in the therapeutic efficacy of platelet concentrate transfusions, and also for the induction of RAEs (146). The BRMs contained in platelet concentrates are also transfused to the recipient, in addition to blood platelets.

Among these molecules, sCD40L has been described as partly responsible for Febrile Non-Haemolytic Transfusion Reactions (FNHTR) after platelet transfusions (147, 148). In addition to its role in inflammation, CD40L appears to be involved in RAEs. Indeed, sCD40L is present in platelet concentrates and its concentration increases during storage (144). Numerous studies have shown that sCD40L is involved in reactions after PC transfusion (28, 149, 150). We have also shown that other soluble factors, such as IL27 and sOX40L, were involved in FNHTR (151, 152). Several soluble factors with a high predictive value for the occurrence of RAEs were identified by mathematical machine learning models, such as sCD40L, IL13 and MIP1α (153). Indeed, this study shows a correlation between the concentration of sCD40L and IL13 and the occurrence of RAEs. Furthermore, MIP1α present in RAE-inducing supernatants seems capable of distinguishing the type of RAE, FNHTR or allergies, depending on its concentration.

In this study, 9,206 platelet concentrate samples were collected at the time of preparation, of which 2,850 were sampled on the day on which the platelet concentrates were delivered. The concentration of sCD40L was assessed for all these samples. In conjunction with haemovigilance services, we collected 140 PCs that had induced RAEs. A mathematical model identified a threshold above which there was a significant association between sCD40L levels and RAEs, allowing entropy to be removed from a large data set. These results indicate that sCD40L in PCs is not always responsible for pathogenicity in patients (RAEs). The thresholds are more discriminating for PPCs than for APCs (154).

Platelet storage lesions are referred to as structural and biochemical changes in platelets. These storage lesions depend on production methods, additive solutions, method of pathogen inactivation, and storage time, etc. Sut C et al. (155) investigated the concentration of sCD40L and sCD62P in apheresis platelet concentrates (APCs) and buffy coat pooled platelet concentrates (PPCs). Nearly 9,000 samples were studied, depending on preparation and storage period. Soluble factors were quantified in platelet supernatants using the Luminex technique. APCs appeared to be more activated than PPCs at the end of the preparation stage, i.e. before storage. However, soluble proinflammatory factors, including sCD40L, are higher during storage in PPCs compared to APCs. Our data highlight the importance of processing and storing platelet concentrates (155). In a study by Sut C et al., nearly 4,000 samples of apheresis platelet concentrates were investigated for donor-related parameters in addition to the preparation and storage processes. Soluble CD40L and CD62P were quantified in platelet concentrate supernatants after preparation and during storage, using Luminex technology. We noted an increase in soluble factors over time. However, the different parameters studied in relation to either the donors or the donations, such as: i) the donor’s gender, ii) the donor’s blood type, iii) the time of collection and iv) the type of apheresis separator, do not appear to significantly impact the platelet activation state and the release of soluble CD40L and CD62P (156).

Post-transfusion dyspnoea (TRALI - Transfusion‐Related Acute Lung Injury) is acute respiratory distress occurring within 24 hours of transfusion. TRALI has a very complex pathogenesis involving various inflammatory cells, such as neutrophils, platelets and endothelial cells (157–160). Some even suggest the involvement of other cells, such as monocytes or (161) pulmonary macrophages (162). Although no consensus has been reached as to which cells are actually involved (neutrophils vs. monocytes vs. pulmonary macrophages vs. blood platelets), there is a common denominator between all these cells, namely the involvement of the CD40L/CD40 protein complex. This immune relationship actively participates in establishing innate and adaptive immunity and, more generally, inflammation (29). By targeting this protein complex, we assumed inhibition of the onset of TRALI, characterised by an unregulated inflammatory state, induced in a murine model by successive injections of LPS and anti-CMH I antibodies. Rather than cell activation, the migration of neutrophils and blood platelets from the vascular compartment to the alveolar space was inhibited. Indeed, in the blood compartment, inflammation is not limited during treatment with neutralising anti-CD40L antibodies, but the action of neutrophils and platelets in the lung is perceptibly reduced. Inhibition of the CD40L/CD40 relationship in this study also appears to have an impact on monocytes. This particular parameter warrants further investigation. This study highlights the long and underestimated role of the CD40L/CD40 protein complex in the pathophysiology of TRALI (163).

TRALI is considered to be one of the post-transfusion reactions with the highest mortality rate (164). This condition, as seen previously, is characterised by severe pulmonary repercussions, which explains the high mortality rate. The pathogenesis of TRALI is similar to that of several inflammatory diseases, such as pancreatitis or inflammatory bowel syndrome. A similar mechanism is involved in pancreatitis - duly orchestrated by the migration of neutrophils into the damaged tissue (165). We assumed that deeper organs, such as the pancreas, may be a secondary target during the onset of TRALI. As both diseases, namely pancreatitis and TRALI, are closely linked, we investigated the involvement of the CD40L/CD40 protein interaction in the regulation of pancreatitis immediately following the onset of TRALI. Using an animal model of TRALI induced by successive injection of LPS and anti-CMH I antibodies and preventive treatment based on anti-CD40L antibodies, we were able to demonstrate that the pancreatic damage observed in our ALI model is prevented to a significant extent when the CD40L/CD40 immune complex is inhibited (166).

## Conclusion and Future Directions

Given i) the large blood platelet count, taking all vascular cellular elements into consideration, ii) their wide range of immune receptors, iii) the ability of platelets to interact with/promote immune cells and endothelial cells, and iv) their active participation in immunity and inflammation, blood platelets have been the subject of numerous investigations into the physiological processes related to inflammation. As a general rule, all of the studies presented in this review show that platelets are capable of covering most of the stages of inflammation, primarily through the CD40L/CD40 interaction, thus confirming their own role in this pathophysiological condition.

Several original reviews have studied “platelet physiology” as an immune cell concept (143, 167), and a significant number of articles (17, 69, 168–172) have recently confirmed this idea. It is now clear that, in addition to their role in haemostasis and thrombosis, platelets have a wide range of other functions. These include key roles in the inflammatory process and immune responses (10, 143, 173). Future research will focus on the critical role of platelets as an immune cell in the host immune response. The key message of this review is that platelets are fully involved in inflammatory processes, particularly *via* the CD40L/CD40 complex, in relation to both exogenous and endogenous stress. Future challenges for therapeutic intervention in disease processes will be to identify medicinal products that block specific targets involved in the complex contribution of platelets to inflammation/immunity without affecting their haemostatic function.

## Author Contributions

FC, HH-C wrote the manuscript. AD, EA-C, TE, CA, AP, ME, PM, OG, and LB revised the manuscript. All authors contributed to the article and approved the submitted version.

## Funding

This work was supported by grants from the Etablissement Français du Sang (EFS)-French National Blood Establishment- and the Association “Les Amis de Rémi” Savigneux, France.

## Conflict of Interest

The authors declare that the research was conducted in the absence of any commercial or financial relationships that could be construed as a potential conflict of interest.

## Publisher’s Note

All claims expressed in this article are solely those of the authors and do not necessarily represent those of their affiliated organizations, or those of the publisher, the editors and the reviewers. Any product that may be evaluated in this article, or claim that may be made by its manufacturer, is not guaranteed or endorsed by the publisher.
